# Energy Efficient Probabilistic Switching ON–OFF Operation in Multiantenna Cooperative Wireless Sensor Networks

**DOI:** 10.3390/s21092937

**Published:** 2021-04-22

**Authors:** Eduardo Yabcznski, Glauber Brante, Richard Demo Souza, Samuel Montejo-Sánchez

**Affiliations:** 1 Graduate Program in Electrical and Computer Engineering, Federal University of Technology (UTFPR), Paraná 80230-901, Brazil; eduyab@alunos.utfpr.edu.br; 2 Department of Electrical and Electronic Engineering, Federal University of Santa Catarina (UFSC), Santa Catarina 88040-900, Brazil; richard.demo@ufsc.br; 3 Programa Institucional de Fomento a la I+D+i, Universidad Tecnológica Metropolitana (UTEM), Santiago 8940577, Chile; smontejo@utem.cl

**Keywords:** cooperative wireless sensor network, multiantenna schemes, ON–OFF probability, amount of information

## Abstract

The effective deployment of Internet of Things (IoT) applications such as smart cities, smart farming and smart transport systems must ensure the network robustness, scalability and longevity. Therefore, guaranteeing the successful delivery of information and extending the lifetime of the nodes that make up a wireless sensor network (WSN) are two essential aspects for IoT applications. This work evaluates the performance of a cooperative WSN by adopting two multiantenna schemes: antenna selection (AS) and beamforming transmission using the singular value decomposition (SVD) technique. In addition, cooperation is established according to an ON–OFF probability, so that the RF receiving circuits of the relays are activated in a probabilistic way, aiming at reducing the energy consumption of the sensors, extending their useful lifetime. Our main goal is to increase the amount of information effectively transmitted by the network, keeping an outage probability constraint. The results show that, when both techniques are used, there is a significant gain in the amount of information effectively transmitted by the network, with emphasis on the AS scheme at short transmission distances. By increasing the number of antennas, it was found that a lower ON–OFF probability is required, i.e., a trade-off is established between the nodes’ hardware complexity and their need for cooperation.

## 1. Introduction

The demand for Internet of Things (IoT) applications is continuously growing. According to Galov [1], around 35 billion IoT devices are in use worldwide by 2021, a figure that is expected to jump to 125 billion by 2030, with over 40% of IoT devices to be used in manufacturing or industry. One of the most desired features for IoT devices is that the energy consumption is as low as possible, extending the sensors useful life as battery replacement is usually unpractical [2]. The use of multiple antennas to exploit spatial diversity is one of the most common approaches to reduce the transmit power consumption, as the power amplifier is one of the most power hungry elements in the radio frequency (RF) chain [3]. Multiple-input multiple-output (MIMO) schemes allow reducing the transmit power while keeping the same outage probability performance when compared to a single-input single-output (SISO) scheme [4]. Nevertheless, despite the benefits in terms of reducing transmit power, increasing the number of antennas also increases the number of RF chains, therefore also increasing the energy consumption of the other electronic circuit elements. As an alternative or even a complement to MIMO techniques, spatial diversity can also be achieved through cooperative communications [5]. In a cooperative transmission, the source node broadcasts its message in a first phase, while nearby nodes act as potential relays in a second phase. Thus, the information reaches the destination node through multiple paths (with different fading realizations), reducing the outage probability.

Energy efficiency in wireless sensor networks (WSNs) has been widely studied in the literature [6,7]. Notwithstanding, due to the constant growth of IoT networks, this subject has received renewed interest, especially considering the optimization of the sleep period of sensor nodes in a network-wide approach (e.g., [8,9,10,11,12]). For instance, to extend network lifetime without compromising performance, the authors of [8] proposed a cooperative scheme, where the nodes are equipped with only one ominidirectional antenna and the receiving circuitry of each relay is switched ON or OFF according to an ON–OFF probability. This probability takes into account system requirements and the relative position of the sensor node with respect to the gateway. Then, to determine the optimal ON–OFF probability, the authors elaborated an optimization problem to maximize the amount of information effectively transmitted by the network, given a quality of service (QoS) constraint.

The authors of [9] proposed a distance-based dynamic duty-cycle allocation algorithm in order to regulate the duty-cycle of the child nodes within a cluster. The nodes closest to the cluster head transmit with larger duty-cycles, since they require less transmit power, while the nodes farther away from the cluster head transmit with smaller duty-cycles. The results show that the proposed algorithm consumes less power than other techniques in the literature, increasing the network lifetime. However, at the cost of increasing the network latency.

Moreover, the characteristics of the carrier sense multiple access with collision avoidance (CSMA-CA) protocol are explored in [10] in order to adjust the duty-cycle of the nodes in a IEEE 802.15.4 network. First, the controller estimates the amount of energy remaining in the nodes, so that, if it is below a specified threshold, the controller reduces the duty-cycle of that node. As a result, network lifetime is extended since nodes stay in sleep state for longer periods. In addition, a fuzzy logic approach was considered by Collotta et al. [11] for both IEEE 802.15.4 and WirelessHART technologies. By processing the remaining battery level, the throughput-to-workload ratio and the link quality, the fuzzy controller is responsible for adjusting the sleeping time and the transmit power of the devices. Simulation and experimental results show an increase of 26% in the lifetime of the IEEE 802.15.4 devices and 40% in the WirelessHART devices, compared to the usual case of setting the sleeping time equal to the sampling period and transmitting with fixed power.

Furthermore, Hammood et al. [12] monitored a patient’s health using an IEEE 802.15.6-based cooperative protocol. The proposed approach uses two coordinating nodes, one on-body and the other outside the body. Then, in the first phase, the sensors broadcast for both coordinators, while a cooperative phase occurs only if the coordinator outside the body does not receive the information correctly, and the retransmission is made by the on-body coordinator. The proposed algorithm minimizes the number of sensor retransmissions, reducing the BER, which directly affects the duty-cycle and consequently the average transmit power of the nodes. As a result, the energy efficiency increases, while the BER is reduced four times compared to a direct transmission approach.

Other complementary approaches, not focusing directly onto the communication aspects, can be found in [13,14]. Shu et al. [13] considered a wireless network sensing area of interest. The objective is to maximize the amount of time that the area is continuously sensed given the constrained energy budget of the wireless nodes. Moreover, the authors assumed that the sensing coverage of multiple nodes may overlap and that wireless energy charging is possible. Then, they proposed to activate different sets of nodes at different times in order to equalize the energy consumption and improve lifetime. Moreover, an energy replenishment strategy is also proposed. However, their focus is solely on the sensing aspect, not on the communication between nodes and to the sink, which is the focus of this work. Furthermore, in [14], a machine learning method is proposed to compensate for missing sensor data, assuming that there is some correlation on different sensors measurements due to spatial proximity. As mentioned by the authors, such approach has very important applications in practice, since adequate data reconstruction can be carried out even when a significant number of sensors is lost.

In this work, we aim at increasing the energy efficiency of an IoT network composed by sensor devices. Differently from the works in [13,14], in this work, we focus on the energy spent at the communication process, not at the sensing tasks. More specifically, we resort to multiantenna cooperative approaches, namely the antenna selection (AS) and transmission beamforming using the singular value decomposition (SVD) technique. We remark that ever smaller antennas are being developed for IoT devices [15], which justifies our investigation. In particular, we exploit the fact that wireless sensor nodes usually employ duty-cycles in order to save energy, so that their circuits switch constantly between active and sleeping states. However, unlike the authors of [9,10,11,12], we do not consider the duty-cycle of a particular MAC layer standard, such as IEEE 802.15.4 [10,11], WirelessHART [11] or IEEE 802.15.6 [12]. On the other hand, we consider cooperative and multiple antenna techniques at the sensor nodes, which considerably changes the optimization of the ON–OFF periods depending on the employed transmission scheme. In addition, we also consider that nodes may optimize their transmit power and adapt their duty-cycles according to groups, depending on the distance with respect to the gateway. Thus, different duty-cycles are spread along the network, which is different from the authors of [9,10,11,12] who considered the same duty-cycle for every node in the network. In addition, these results can be adapted to any particular MAC layer standard for a system implementation. A summary of the differences between this work and the most relevant related work is presented in Table 1.

Therefore, we establish an ON–OFF probability for the reception RF circuit chain based on [8], with the goal of increasing the amount of information flowing in the network, i.e., the total number of bits transmitted by the sensors during their lifetime. However, differently from the work in [8], we consider the use of multiple antennas at the sensor nodes as well as power control. Then, we maximize the sleeping states of the devices in order to respect a required outage probability constraint. As a consequence, cooperation occurs based on this optimized ON–OFF probability, which depends on the total number of sensors in the communication range, number of antennas and employed MIMO technique. Such framework is considerably different from the literature, so that results show a significant increase in the amount of information effectively transmitted by the network, with the AS technique being particularly important in this scenario. In addition, with a larger number of antennas, the results show that a lower cooperation probability is required, providing a considerable gain for AS and a degradation in SVD. Moreover, we also show that there is an optimal transmit power that maximizes the amount of information, depending on the network area. Then, we employ a sequential quadratic programming (SQP) to solve the joint optimization of the transmit power and the ON–OFF probability, yielding a considerable gain in the amount of information effectively transmitted compared to the scenario with fixed transmit power. The numerical results demonstrate the importance of power control and multiple antennas in order to achieve relevant gains in terms of information transmitted in the network with respect to that achieved in [8].

The remainder of this paper is organized as follows. Section 2 presents the system model. Section 3 presents the probabilistic switching ON–OFF cooperation scheme, while Section 4 defines the employed evaluation metrics: the amount of information and the energy consumption. Next, Section 5 gives some numerical examples and Section 6 concludes the paper.

## 2. System Model

**Figure 1:**
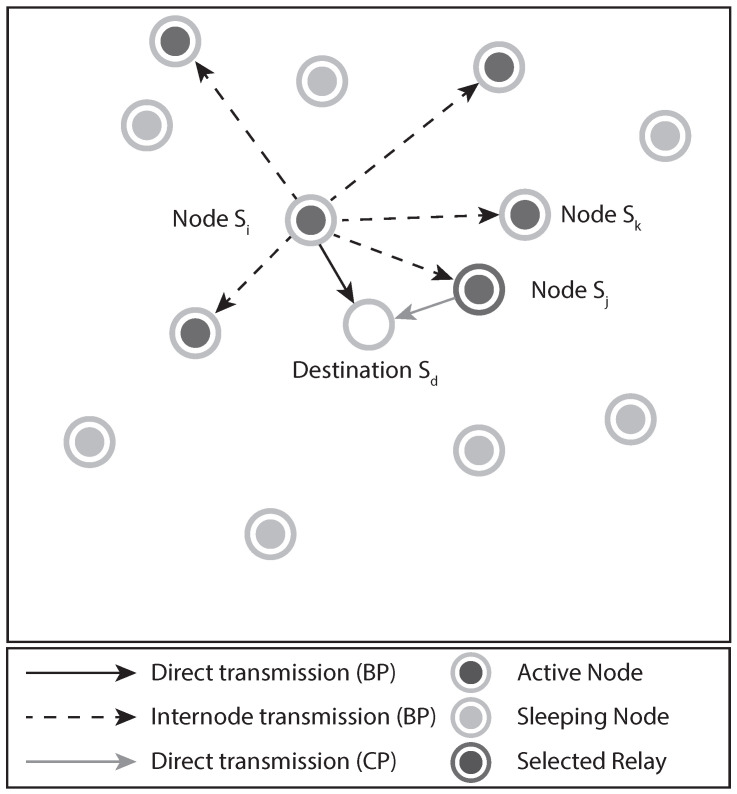
System model showing a broadcast transmission from node $S_{i}$, targeting the destination node $S_{d}$, with retransmission from the relay node $S_{j}$.

We assume a wireless network, as illustrated by Figure 1, where *M* sensor nodes are distributed in a square area of $D\times Dm^{2}$, trying to communicate with a destination node located at the center. Every node can cooperate with each other using the incremental decode-and-forward (IDF) protocol [5]. Then, a given sensor node denoted by $S_{i}$, i∈S={1,…,M}, transmits its information to the destination $S_{d}$ during the first time-slot, which we denote by broadcast phase (BP). Furthermore, we assume that the sensors transmit using time division multiple access (TDMA) in order to avoid collisions.

If the destination is unable to decode the transmission from the source, it indicates the failure of the direct transmission by broadcasting a NACK message through an error-free feedback channel. Then, any sensor node can act as a relay in the second time-slot (note that, if no NACK is broadcast, then this time-slot can be used by another sensor node in the next BP), which we denote as cooperative phase (CP). A sensor node $S_{j}$, j∈S, is eligible to act as a relay if it has decoded the message from $S_{i}$ correctly in the BP and continues switched ON during the transmission of the NACK. We assume that the channel state information (CSI) between each relay and $S_{d}$ can be estimated by the NACK message transmitted through the feedback channel. Let us define the set of the nodes that successfully decoded the message from $S_{i}$ during the BP by $S^{*}$. Then, the relay with the highest SNR with respect to the destination in $S^{*}$ can be chosen during the CP. In addition, we also consider that the source node itself can retransmit during the CP if the conditions are more favorable, and only one node acts as a relay. For instance, as proposed in [16], each node able to retransmit can employ its own timer before transmitting inversely proportional to the SNR of the received NACK signal.

### 2.1. Channel Model

We assume that all nodes have the same hardware, being equipped with *n* antennas (nevertheless, the analysis can be easily extended to the case where each node has a different number of active antennas), of which $\hat{n}$ are active, such that $\hat{n}\leq n$. Then, the received signal in a transmission between any two nodes is given by

(1) $$y_{ik,\varphi }=\sqrt{\frac{\kappa_{ik}P_{i,\varphi }}{\hat{n}}}H_{ik,\varphi }x+w_{k,\varphi },$$

where i∈S represents the source node and $k\in \left\{S\;\cup \{d\}\right\}$ represents the union of the potential relays with the destination node, acting as the receiver. Moreover, φ∈{BP,CP} represents the broadcast or cooperation phases, $P_{i,\varphi }$ is total transmit power of node $S_{i}$ during phase φ, $\kappa_{ik}$ is the path loss and $H_{ik,\varphi }$ is the $\hat{n}\times \hat{n}$ matrix of the channel coefficients, whose elements $h_{ik,\varphi }$ are independent and identically distributed random variables with quasi-static Nakagami-*m* distribution. Next, x is the $\hat{n}\times 1$ message vector, with unit energy, and $w_{k,\varphi }$ is the $\hat{n}\times 1$ additive white Gaussian noise (AWGN) vector at the receiver, with variance $N_{0}/2$, where $N_{0}$ is the noise power spectral density.

The path-loss is assumed to be [17]

(2) $$\kappa_{ik}=\frac{c^{2}}{{\left(4\pi f_{c}\right)}^{2}d_{ik}^{\alpha }},$$

where *c* is the light speed in vacuum, $f_{c}$ is the carrier frequency, $d_{ik}$ is the distance between nodes $S_{i}$ and $S_{k}$ and α is the path-loss exponent. The instantaneous SNR in the link between nodes $S_{i}$ and $S_{k}$ is

(3) $$\gamma_{ik,\varphi }={∥H_{ik,\varphi }∥}_{F}^{2}\cdot {\bar{\gamma }}_{ik,\varphi },$$

where ${∥\cdot ∥}_{F}$ is the Frobenius norm [18]. Since the fading follows a Nakagami-*m* distribution, the coefficients ${\left|h_{ik,\varphi }\right|}^{2}$ follow a Gamma distribution with parameter *m*. Moreover, the average SNR per receive antenna is given by

(4) $${\bar{\gamma }}_{ik,\varphi }=\frac{\kappa_{ik}P_{i,\varphi }}{\hat{n}N_{0}B},$$

where *B* is the system bandwidth.

### 2.2. Link Outage Probability

In our analysis, we consider two MIMO schemes, AS and SVD, while SISO is used for comparison purposes. Then, in the following, we define the link outage probabilities, i.e., between any two nodes, for each of these transmission schemes. Following the Shannon limit, we define $\gamma_{0}=2^{R_{0}/B}-1$ as the SNR threshold for decoding, where $R_{0}$ is the bit rate, so that the link outage probability between nodes $S_{i}$ and $S_{k}$ is $p_{ik,\varphi }=\mathrm{Pr}\left\{\gamma_{ik,\varphi }<\gamma_{0}\right\}$.

#### 2.2.1. Single-Input Single-Output (SISO)

In this scheme, we assume the network sensor nodes with only one antenna, $n=\hat{n}=1$. Then, in Nakagami-*m* fading channels, the link outage probability is [17]

(5) $$p_{ik,\varphi }^{\left(\mathrm{SISO}\right)}=1-\frac{\Gamma (m,m\gamma_{0}/{\bar{\gamma }}_{ik,\varphi })}{\Gamma (m)},$$

where $\Gamma \left(x\right)=\int_{0}^{\infty }e^{-t}t^{x-1}dt$ is the complete Gamma function and $\Gamma \left(a,x\right)=\int_{a}^{\infty }e^{-t}t^{x-1}dt$ is the upper incomplete Gamma function [19].

#### 2.2.2. Antenna Selection (AS)

In the AS scheme, we assume that the nodes have *n* antennas, but only $\hat{n}=1$ antenna is active at both transmitter and receiver. With the goal of selecting the pair of antennas yielding the highest SNR, we consider that a few pilot symbols are sent by each transmit antenna, so that the receiver estimates the CSI between all antennas. Then, the antenna index for the transmitter is fed back through an error-free feedback channel (let us remark that the energy cost for transmission/reception of the feedback bit is not considered, as these packets are very short compared to the packets conveying information) as in a transmit antenna selection scheme [20], while the receiver keeps only the best antenna (with respect to the transmit antenna) active. As a result, smaller energy consumption is obtained since only one RF chain remains active at each side. Let us remark that the use of multiple antennas in a transmission scheme implies in multiple RF chain, whose energy consumption is defined in Section 4.2.

Then, the link outage probability, both BP and CP, is given by [21]

(6) $$p_{ik,\varphi }^{\left(\mathrm{AS}\right)}={\left(1-\frac{\Gamma (m,m\gamma_{0}/{\bar{\gamma }}_{ik,\varphi })}{\Gamma (m)}\right)}^{{\hat{n}}^{2}}.$$

The sole exception is from the relay point of view during the BP. Since the transmit antenna selection is made for the source-destination link, this selection is seen as a random event by the relay. Thus,

(7) $$p_{ij,\mathrm{BP}}^{\left(\mathrm{AS}\right)}={\left(1-\frac{\Gamma (m,m\gamma_{0}/{\bar{\gamma }}_{ij,\mathrm{BP}})}{\Gamma (m)}\right)}^{\hat{n}},$$

where j∈S represents the relay node, as illustrated by Figure 1.

#### 2.2.3. Singular Value Decomposition (SVD)

In this scheme, we assume that the sensors employ all *n* antennas in a beamforming fashion through a SVD scheme. In this case, the link outage probability for BP and CP is [20]

(8) $$p_{ik,\varphi }^{\left(\mathrm{SVD}\right)}\approx \left(1-\frac{\Gamma (m,mn\left(2^{R_{0}/\left(Bn\right)}-1\right)/{\bar{\gamma }}_{ik,\varphi })}{\Gamma (m)}\right)\sum_{q=0}^{n^{2}-1}\frac{1}{q!}{\left(\frac{n(2^{R_{0}/\left(Bn\right)}-1)}{{\bar{\gamma }}_{ik,\varphi }}\right)}^{q}.$$

Similar to the AS scheme, the beamforming vector is designed for the source-destination link only, so that the link outage probability between source and relay in the BP is

(9) $$p_{ij,\mathrm{BP}}^{\left(\mathrm{SVD}\right)}=\left(1-\frac{\Gamma (m,m\gamma_{n}/{\bar{\gamma }}_{ij,\mathrm{BP}})}{\Gamma (m)}\right)\sum_{q=0}^{n-1}\frac{1}{q!}{\left(\frac{\gamma_{n}}{{\bar{\gamma }}_{ij,\mathrm{BP}}}\right)}^{q},$$

which assumes the use of MRC at the relay.

## 3. Probabilistic Switching ON–OFF Cooperation

As mentioned above, each cooperative node $S_{j}$ is able to estimate its own CSI using the NACK message from the destination node in the CP. Thus, in possession of $\gamma_{jd,\mathrm{CP}}$, $S_{j}$ waits for a time $t_{j}\propto 1/\gamma_{jd,\mathrm{CP}}$ before transmitting. In addition, when a retransmission occurs, we assume a CSMA-CA protocol, so that collision is not considered in our analysis. Then, the node with the highest SNR with respect to the destination (including the source) will be the first to retransmit.

In this work, we employ a probabilistic switching ON–OFF cooperation (PSwC) strategy [8], so that each sensor has a probability of being awake or asleep, which we define as the ON–OFF probability denoted by $\xi_{ij}$. Such probability determines if the sensor $S_{j}$ is eligible to act as a relay to $S_{i}$ in a given time-slot or not. In practice, each network node generates a random number $\nu_{j}=U\left(0,1\right)$, uniformly distributed between zero and one, which is compared to $\xi_{ij}$. If $\nu_{j}<\xi_{ij}$, and then $S_{j}$ becomes active (switch ON) and can cooperate with $S_{i}$. Conversely, when $\nu_{j}\geq \xi_{ij}$, $S_{j}$ turns to sleep mode (switch OFF) until the $S_{i}$ transmission time-slots ends.

By taking the ON–OFF probability into account, the link outage probability in the BP becomes

(10) $${\tilde{p}}_{ij,\mathrm{BP}}^{\left(\mathrm{sch}\right)}=1-\xi_{ij}\left(1-p_{ij,\mathrm{BP}}^{\left(\mathrm{sch}\right)}\right),$$

with (sch)∈{SISO,AS,SVD}.

Then, the total outage probability $P_{i}^{\left(\mathrm{sch}\right)}$ is defined as the probability that the message transmitted by $S_{i}$ is not correctly decoded after both direct transmission and cooperation. A packet loss occurs when: (i) communication between $S_{i}$ and $S_{d}$ fails in the BP and the communications between the relay and $S_{d}$ fails in the CP; or (ii) when $S_{i}$ fails in the BP to communicate with any other node. Since only one relay is active, the total outage probability is given by [8]

(11) $$\begin{array}{cc} P_{i}^{\left(\mathrm{sch}\right)} & ={\left(p_{id,\mathrm{BP}}^{\left(\mathrm{sch}\right)}\right)}^{2}\prod_{j}\left[{\tilde{p}}_{ij,\mathrm{BP}}^{\left(\mathrm{sch}\right)}+p_{jd,\mathrm{CP}}^{\left(\mathrm{sch}\right)}(1-{\tilde{p}}_{ij,\mathrm{BP}}^{\left(\mathrm{sch}\right)})\right]\\ \; & ={\left(p_{id,\mathrm{BP}}^{\left(\mathrm{sch}\right)}\right)}^{2}\prod_{j}\left[1-\xi_{ij}\left(1-p_{ij,\mathrm{BP}}^{\left(\mathrm{sch}\right)}\right)\left(1-p_{jd,\mathrm{CP}}^{\left(\mathrm{sch}\right)}\right)\right]. \end{array}$$

Another important parameter is the probability that the node $S_{j}$ acts as relay to retransmit the message from the source node $S_{i}$, which we denote by $\rho_{ij}$ and define as the retransmission probability. Let us define the set of the nodes that successfully decoded the message from $S_{i}$ during the BP by $S^{*}$, which includes the source node itself. Then, the node $S_{j}$ acts as relay if it belongs to $S^{*}$, and if its SNR with respect to the destination is higher than all other nodes in $S^{*}$, i.e., if $\gamma_{jd,\mathrm{CP}}>\max_{l\in S_{-j}^{*}}\left\{\gamma_{ld,\mathrm{CP}}\right\}$, where $S_{-j}^{*}$ is a subset of $S^{*}$, excluding the node $S_{j}$. Then, following Bordón et al. [8],

(12) $$\rho_{ij}=\sum_{S_{-j}^{*}}\mathrm{Pr}\left\{\max_{l\in S_{-j}^{*}}\left\{\gamma_{ld,\mathrm{CP}}\right\}<\gamma_{jd,\mathrm{CP}}\right\}\mathrm{Pr}\left\{S_{-j}^{*}\right\},$$

where $\mathrm{Pr}\{S_{-j}^{*}\}$ is the probability that the nodes whose indexes belong to $S_{-j}^{*}$ can successfully decode the message from the source in the BP, while all other nodes that do not belong to the $S_{-j}^{*}$ cannot. Finally, the retransmission probability can be obtained as [8]

(13) $$\rho_{ij}=\int_{0}^{\infty }\rho_{ij}\left(x\right)\frac{{\left(m/{\bar{\gamma }}_{jd,\mathrm{CP}}\right)}^{m}x^{m-1}}{\Gamma \left(m\right)e^{mx/{\bar{\gamma }}_{jd,\mathrm{CP}}}}dx,$$

where $\rho_{ij}\left(x\right)=\prod_{l\in S_{-j}^{*}}\left[1-(1-p_{il}^{\left(\mathrm{sch}\right)})\mathrm{Pr}\left\{\gamma_{ld,\mathrm{CP}}<x\right\}\right]$.

## 4. Amount of Information Transmitted and Energy Consumption

### 4.1. Amount of Information

In this work, we employ the amount of information as the performance metric, which is defined as the number of bits of information successfully received by the destination node, until the first node in the network exhaust its battery power. Assuming that $N_{T}$ is the number of messages transmitted by node $S_{i}$ during the network lifetime and that each message has a fixed length of *L* bits, the amount of information transmitted in the network, in bits, is defined as

(14) $$B_{T}^{\left(\mathrm{sch}\right)}=L\;N_{T}\sum_{i\in S}\left(1-P_{i}^{\left(\mathrm{sch}\right)}\right).$$

### 4.2. Energy Consumption Model

At the transmitter, we assume that the total transmit power of the node $S_{i}$, $P_{i,\varphi }$, is equally divided by each active antenna, i.e., the transmit power per antenna is equal to $P_{i,\varphi }/\hat{n}$. Thus, the energy consumed by the node $S_{i}$ during the transmission of one message is

(15) $$e_{t}=\frac{(P_{i,\varphi }\;\eta^{-1}+P_{\mathrm{ct}}\;\hat{n})L}{R_{0}},$$

where η is the power amplifier efficiency and $P_{\mathrm{ct}}$ is the power consumed by each transmission circuit chain [3].

Conversely, the energy consumed during reception of one message is

(16) $$e_{r}=\frac{(P_{\mathrm{cr}}\hat{n})L}{R_{0}},$$

where $P_{\mathrm{cr}}$ is power consumed by the circuits at the receiver.

Then, the total energy consumed by node $S_{i}$ to transmit $N_{T}$ messages is given by

(17) $$E_{i}=E_{i,t}+E_{i,r}+E_{i,c},$$

where $E_{i,t}=N_{T}e_{t}$ is the energy consumed to transmit during the BP; $E_{i,r}=N_{T}\sum_{j}\xi_{ji}e_{r}$ is the energy consumed when receiving the message from other nodes $S_{j}$, also during BP; and

(18) $$E_{i,c}=N_{T}e_{t}p_{id,\mathrm{BP}}^{\left(\mathrm{sch}\right)}\rho_{ii}+N_{T}\sum_{j}\xi_{ji}e_{t}\rho_{ji}p_{jd,\mathrm{BP}}^{\left(\mathrm{sch}\right)}(1-p_{ji,\mathrm{BP}}^{\left(\mathrm{sch}\right)})$$

is the energy consumed to transmit as a relay in the CP, with the first term in $E_{i,c}$ denoting the case when $S_{i}$ retransmits its own message during the CP, while the summation term denotes the case when $S_{i}$ acts as a relay for any other node $S_{j}$. Rewriting (17), we arrive at [8]

(19) $$\begin{array}{c} E_{i}=N_{T}\left\{e_{t}\left(1+p_{id,\mathrm{BP}}^{\left(\mathrm{sch}\right)}\rho_{ii}\right)+\sum_{j}\xi_{ji}\left[e_{r}+e_{t}\rho_{ji}p_{jd,\mathrm{BP}}^{\left(\mathrm{sch}\right)}(1-p_{ji,\mathrm{BP}}^{\left(\mathrm{sch}\right)})\right]\right\}. \end{array}$$

Considering that $S_{i}$ is the first node to drain all the energy from its battery and assuming the complete battery charge ($E_{max}$) of this node is used, the number of messages transmitted by this node can be written as

(20) $$N_{T}=\frac{E_{max}}{e_{t}+\max_{i}\left\{\Upsilon_{i}\right\}},$$

where $\Upsilon_{i}=e_{t}p_{id,\mathrm{BP}}^{\left(\mathrm{sch}\right)}\rho_{ii}+\sum_{j}\xi_{ji}\left[e_{r}+e_{t}\rho_{ji}p_{jd,\mathrm{BP}}^{\left(\mathrm{sch}\right)}(1-p_{ji,\mathrm{BP}}^{\left(\mathrm{sch}\right)})\right]$, in which it is worth noting that, since the numerator of (20) is fixed (maximum energy contained in a battery), to increase the number of messages transmitted, it is necessary to decrease the denominator ($e_{t}+\max_{i}\left\{\Upsilon_{i}\right\}$).

Note that our main goal is to increase the amount of information effectively transmitted by the network, keeping an outage probability constraint. This purpose can only be achieved by extending the network lifetime, which is ultimately determined by the battery initial charge, which is the same for all nodes, and the use of an efficient cooperation mechanism that allows nodes to save energy when cooperation is not required.

### 4.3. ON–OFF Probability and Transmit Power Optimization

With the goal of reducing the overall power consumption, two variables are optimized in the following proposal, the transmit power and the ON–OFF probability. Such approach is different from that of Bordón et al. [8], in which the transmit power is fixed. The rationale here is to tune the transmission power in order to reduce the energy consumption, so that we increase the amount of information transmitted by the network. We define this problem as a constrained optimization, implying that for every $S_{i}$, i∈S, we have

(21a) $$\begin{array}{cc} P_{\varphi }^{*},\;\xi_{i}^{*} & =\underset{P_{i,\varphi },\xi_{ij}^{*}}{\mathrm{argmax}}\;B_{T}^{\left(\mathrm{sch}\right)} \end{array}$$

(21b) $$\begin{array}{cc} s.t. & \;P_{i}^{\left(\mathrm{sch}\right)}\leq P_{o},\;\forall i, \end{array}$$

(21c) $$\begin{array}{cc} \; & \;0\leq \xi_{ij}\leq 1,\;\forall j, \end{array}$$

(21d) $$\begin{array}{cc} \; & \;P_{\min}\leq P_{i,\varphi }\leq P_{\max}, \end{array}$$

where $\xi_{i}^{*}=\left\{\xi_{ij}^{*}\right\}$ is a vector of *M* elements, containing the optimal relay ON–OFF probability $\xi_{ij}^{*}$ for each sensor node $S_{j}$ operating as a relay to $S_{i}$. Moreover, $P_{\varphi }^{*}$ is the optimal transmit power, assumed to be the same for every sensor $S_{i}$. In addition, there are *M* constraints associated with (21b), ensuring an outage probability threshold $P_{o}$ for every sensor. Similarly, (21c) is also a set of *M* constraints related to $\xi_{ij}$. Finally, $P_{\min}$ and $P_{\max}$ in (21d) denote, respectively, the minimal and the maximal transmit power used by the sensors.

Due to the complexity of the optimization problem, we employ a sequential quadratic programming (SQP) to solve the optimization in (21a). The SQP method is an iterative method for constrained nonlinear optimization [22], readily available in Matlab through the fmincon function.

## 5. Numerical Results

**Figure 2:**
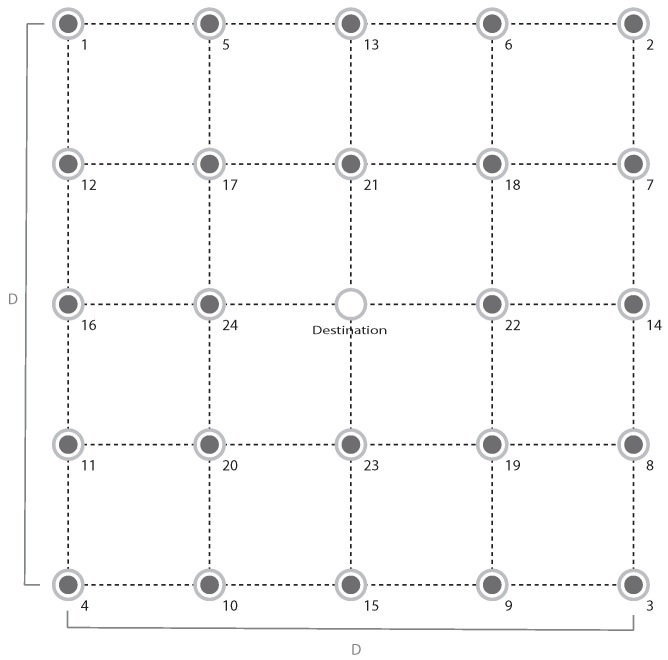
Network containing M=24 sensor nodes distributed and spaced equally in a square area with D=200 m.

This section presents some numerical examples to illustrate the proposed approach. We consider AS and SVD transmission schemes, as well as SISO for comparison purposes. Due to the computational burden to solve the optimization problem, $S_{d}$ is chosen as the central controller since it has greater processing capacity than the other nodes in the network. In addition, as in [8], we consider M=24 nodes symmetrically distributed in space according to Figure 2, in a square region with sides of D=200 m. Then, all sensor nodes employ the same transmit power, so that it is possible to group the sensors into five groups: $S_{g,1}=\left\{1,\ldots ,4\right\}$, $S_{g,2}=\left\{5,\ldots ,12\right\}$, $S_{g,3}=\left\{13,\ldots ,16\right\}$, $S_{g,4}=\left\{17,\ldots ,20\right\}$ and $S_{g,5}=\left\{21,\ldots ,24\right\}$. The above grid deployment is representative of some practical setups, such as precision agriculture or smart buildings. Furthermore, the grid deployment assumption does not limit the application of our proposal, but it considerably simplifies the analysis and understanding. In a more general setup, where nodes are randomly distributed, the strategy would be to group nodes according to their proximity, so that the ON–OFF probabilities of these groups can be optimized in the same way as in the grid deployment. Finally, Table 2 describes the system parameters, following Bordón et al. [8].

### 5.1. Fixed Transmit Power

**Figure 3:**
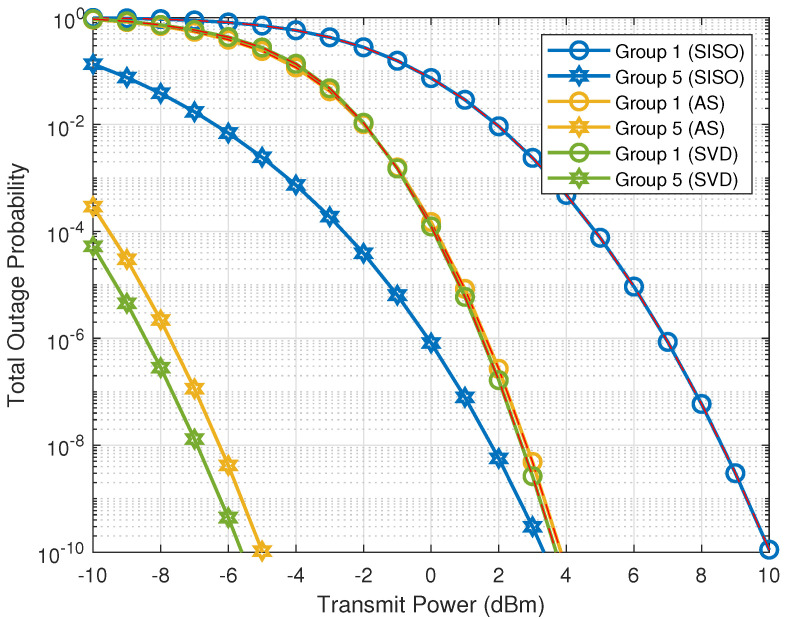
Total outage probability ($P_{i}$) as a function of transmit power with $\xi_{ij}=1$ and n=2 antennas.

First, we consider the case where the transmit power is fixed, i.e., excluded from the optimization problem in (21a). Figure 3 shows the total outage probabilities of SISO, AS and SVD transmission schemes when $\xi_{ij}=1$ and n=2 antennas. To facilitate the visualization only the groups $S_{g,1}$ and $S_{g,5}$ are shown, formed, respectively, by the farthest and closest nodes with respect to $S_{d}$. As we observe, the difference in terms of outage probability for AS and SVD is very small, especially when considering group $S_{g,1}$. Therefore, the outage threshold $P_{o}$ (marked as the dashed red curves in Figure 3) in the optimization is established as the outage probability of group $S_{g,1}$ when $\xi_{ij}=1$, which may be different for each employed transmission scheme. In other words, $P_{o}=\max_{i}\left\{P_{i}\right\}:\xi_{ij}=1$, ∀{i,j}∈S.

**Figure 4:**
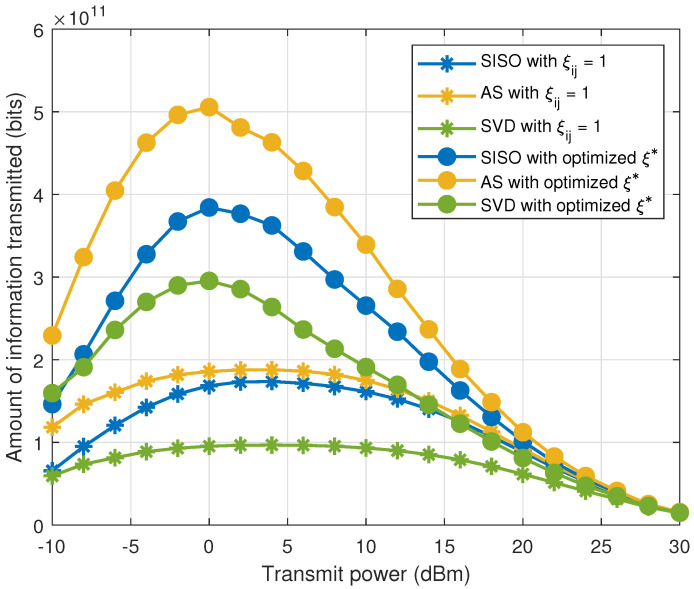
Amount of information transmitted by SISO, AS and SVD with n=2 antennas, comparing the cases with $\xi_{ij}=1$ and optimized $\xi_{i}^{*}$.

Figure 4 shows the amount of information transmitted comparing the cases when the nodes circuits is always ON i.e., with $\xi_{ij}=1$, and after the optimization of $\xi_{i}^{*}$. As we observe, there is a significant increase in the amount of information transmitted by optimizing $\xi_{i}^{*}$, since the relay receiving circuits are not always ON, extending the sensors’ lifetime. In addition, an increase of up to two times in the amount of information transmitted is observed for the AS scheme. In fact, the spatial diversity due to the multiple antennas combined to the optimization of the ON–OFF probability brings expressive gains to the network. The spatial diversity schemes benefit more from the optimization as, due to the increased transmission reliability, more nodes can turn to the OFF state, saving energy. Moreover, with spatial diversity, nodes further from the destination are more able to cooperate with other nodes, balancing the network energy consumption, while, in SISO, nodes closer to the destination tend to cooperate much more often. Furthermore, when comparing spatial diversity schemes, AS uses only one active RF chain per node, while SVD uses one RF chain per antenna. Thus, the combination of lower power consumption (specially for AS) and the spatial diversity gains are essential in this setup, allowing nodes to sleep more frequently, increasing the network lifetime and, consequently, the amount of information. Another important observation is that there is an optimal transmit power for each scheme. By increasing $P_{i,\varphi }$, the outage constraint $P_{o}$ decreases. As a consequence, the ON–OFF probability must increase in order to attain the QoS constraints, increasing the overall energy consumption. Finally, notice also that the optimal transmit power is around 0 dBm when $\xi_{i}^{*}$ is optimized. This clearly highlights the importance of the joint optimization of the ON–OFF probability and the transmit power in order to increase network lifetime and the amount of information transmitted by the network.

**Figure 5:**
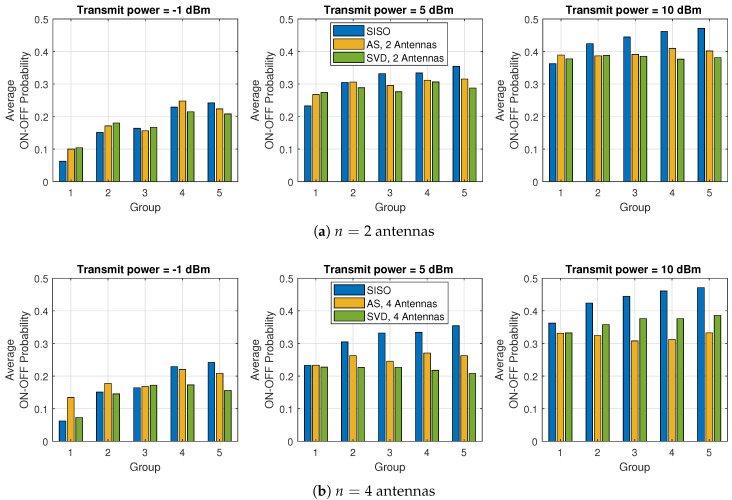
Average value of the ON–OFF cooperative probability for each group, varying the transmit power for SISO, AS and SVD.

Next, we plot the average ON–OFF cooperation probability for each of the five groups of nodes in Figure 5, considering n=2 antennas in Figure 5a and n=4 antennas in Figure 5b, in each sensor node. As mentioned above, when $P_{i,\varphi }$ increases, the $P_{o}$ constraint decreases and the groups tend to cooperate more often. On the other hand, by increasing the number of antennas (comparing Figure 5a with Figure 5b), the ON–OFF probability decreases due to the higher diversity order provided by *n*. In addition, notice also that SISO has a higher impact in terms of the optimization of $\xi_{i}^{*}$ when $P_{i,\varphi }$ increases than AS and SVD. Although SISO is also cooperative and experiences spatial diversity by the use of the relays, the multiantenna schemes have an additional degree of diversity, which impacts the ON–OFF optimization. When the transmission power increases, the outage probability threshold decreases, since $P_{o}=\max_{i}\left\{P_{i}\right\}:\xi_{ij}=1$, ∀{i,j}∈S. This is the reason the cooperation probability increases with the transmit power, which usually demands more from the nodes closer to the destination. Therefore, by increasing the spatial diversity employing multiantenna schemes, we can increase sleep states of the nodes as well as we allow more opportunities for nodes more distant to the destination to cooperate with other nodes.

### 5.2. Optimized Transmit Power

As observed in the previous results, when the nodes transmit power is low, $P_{i}^{\left(\mathrm{sch}\right)}$ in (11) increases, consequently decreasing $B_{T}^{\left(\mathrm{sch}\right)}$. On the other hand, high transmit power implies a very low $P_{o}$, requiring constant relaying, increasing $\xi_{ij}$ and decreasing $N_{T}$ in (20). As a consequence, there is an optimal transmit power to maximize $B_{T}^{\left(\mathrm{sch}\right)}$. Then, in the following, we assume that the power is optimized within the range of [−10,30] dBm, for different sizes of the square area *D*.

**Figure 6:**
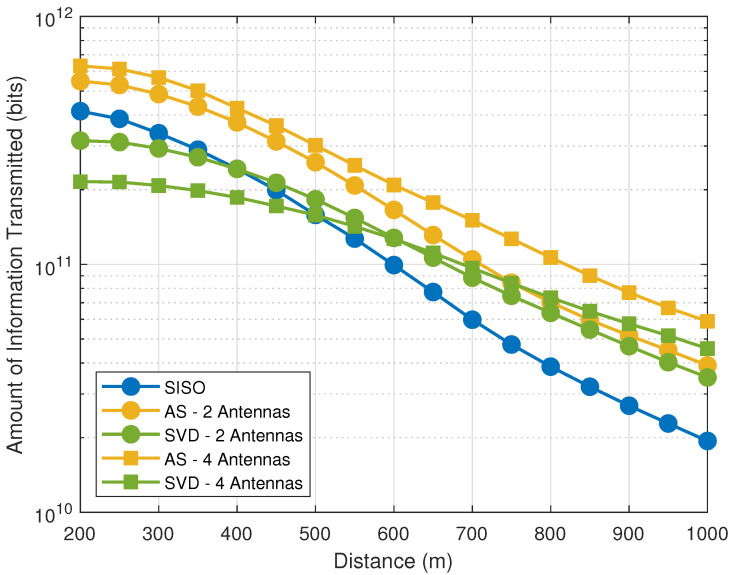
Amount of information with optimized $P_{\varphi }^{*}$ and $\xi_{i}^{*}$ as a function of the side of the squared area (*D*), for SISO, AS and SVD with $R_{0}/B=1$ bps/Hz.

**Figure 7:**
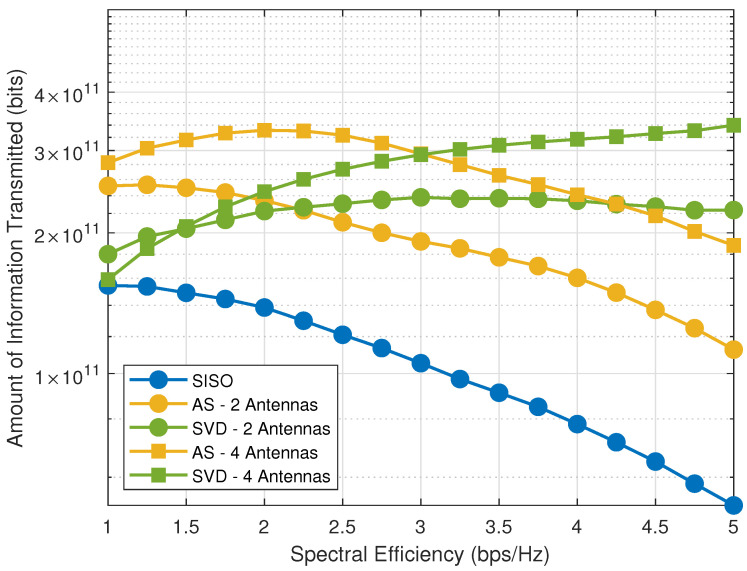
Amount of information with optimized $P_{\varphi }^{*}$ and $\xi_{i}^{*}$ as a function of the spectral efficiency, for SISO, AS and SVD with D=500 m.

Figure 6 plots the amount of information, with optimized $P_{\varphi }^{*}$ and $\xi_{i}^{*}$, as a function of the side of the squared area (*D*). As we observe, the AS scheme outperforms the other schemes is most situations. In a complementary way, Figure 7 investigates the amount of information as a function of the system spectral efficiency, in which we observe that AS performs better for smaller spectral efficiencies (up to 2.2 bps/Hz with n=2 antennas and up to 3 bps/Hz with n=4 antennas), while SVD has increased performance when the spectral efficiency increases.

**Figure 8:**
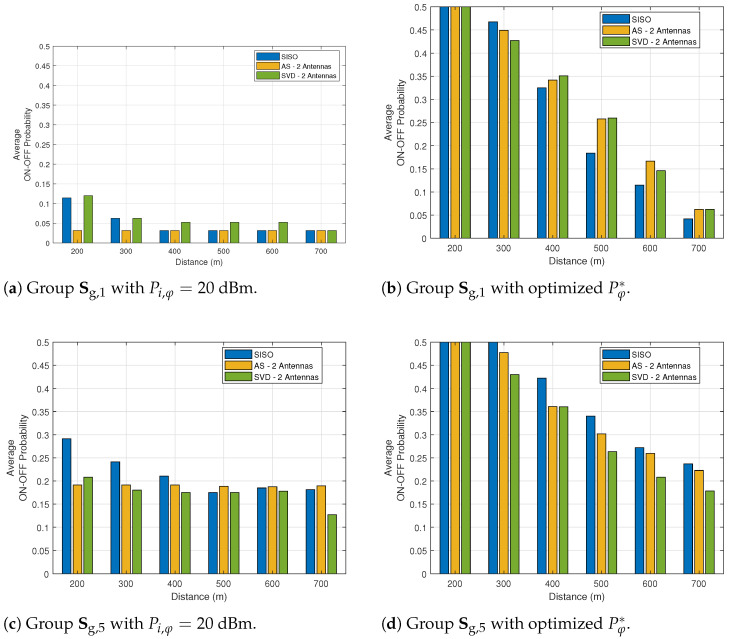
Average ON–OFF cooperation probability as a function of the distance *D* for SISO, AS and SVD with n=2 antennas.

Table 3 shows the relative gain, in terms of amount of information, of the power optimization method with respect to the case of fixed transmit power, with $P_{i,\varphi }=20$ dBm. The optimization of the ON–OFF probability is carried out in both cases. As we notice, for short distances, the gain in selecting the best transmit power for short distances is quite important, with an increase of up to 80% in the number of bits transmitted by the network. This result is complemented by Figure 8, which shows the average ON–OFF probability with fixed and optimized transmit power of groups $S_{g,1}$ and $S_{g,5}$. As we observe, the transmit power allocation considerably decreases the ON–OFF probability, i.e., saving energy from the relays which tend to be in sleep mode more often, increasing the amount of information with the proper power allocation.

**Figure 9:**
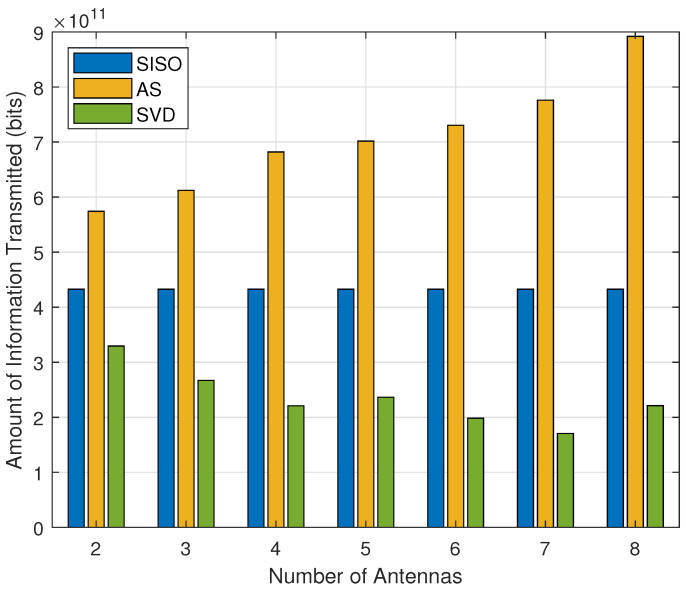
Amount of information as a function of the number of antennas, with optimized $P_{\varphi }^{*}$ and $\xi_{i}^{*}$, for SISO, AS and SVD with $R_{0}/B=1$ bps/Hz.

Finally, Figure 9 analyzes the amount of information as a function of the number of antennas at the sensors, for D=200 m. As observed, the amount of information transmitted by the network always increases with *n* for the AS scheme, regardless of the transmit distance, while, for the SVD scheme, n=2 maximizes the amount of information when D=200 m. Furthermore, when *D* increases, SVD presents an optimal number of antennas that maximizes the amount of information. This is due to the energy consumption of the RF chain required by each antenna, so that AS selects a single pair of antennas to remain active at each transmission, while all RF chains are transmitting/receiving with the SVD scheme, increasing the energy consumption and reducing the network lifetime.

## 6. Conclusions

This study analyzed the performance gain by combining two spatial diversity techniques: multiple antennas and cooperation. We used AS and SVD as the multiple antennas techniques, combined with a probabilistic switching ON–OFF cooperation, where the relays circuits are switched ON or OFF in a probabilistic fashion. Next, we defined the optimization of the ON–OFF probability combined with power allocation in order to maximize the amount of information transmitted by the network. The results show that there is a significant gain in the amount of effective information transmitted by the network by combining the two techniques. For instance, by increasing the number of antennas, the nodes ON probability decreases significantly for the same QoS constraints, thus extending the network lifetime. When transmission power optimization is enabled, the benefits are even greater in terms of amount of information transmitted. In addition, we demonstrated that the AS scheme shows increased performance for shorter communication distances, increasing the amount of information transmitted as the number of antennas increases. On the other hand, SVD performs better when either the transmit distance or the spectral efficiency increases. Finally, we can conclude that AS always benefits from increasing the number of antennas in operation, while, in SVD, the optimal number of antennas depends on the transmission distance. These final conclusions open the expectation of future works that consider a system that can operate with different diversity schemes simultaneously according to the location of its nodes.

## Figures and Tables

**Table 1:** Comparison with related work.

Reference	Duty Cycle	Multiple Antennas	Cooperation	Power Allocation
This Work	Non-Uniform	✓	✓	✓
[**8**]	Non-Uniform	×	✓	×
[**9**]	Uniform	×	×	×
[**10**]	Uniform	×	×	×
[**11**]	Uniform	×	×	×
[**12**]	Uniform	×	×	×

**Table 2:** System parameters [8].

Parameter	Value
Message length	L=50 bits
Transmission rate	$R_{0}=200$ kbps
Noise power spectral density	$N_{0}=-174$ dBm/Hz
System bandwidth	B=200 kHz
Power amplifier efficiency	η=0.35
Nakagami-*m* fading parameter	m=1
Path-loss exponent	α=4
Carrier frequency	$f_{c}=2.5$ GHz
Initial battery charge	$E_{\max}=10$ J
Circuit power consumption	$P_{\mathrm{ct}}=P_{\mathrm{rt}}=10$ dBm

**Table 3:** Relative gain, in terms of amount of information, of the power optimization method with respect to the case of fixed transmit power with $P_{i,\varphi }=20$ dBm with n=2 antennas.

Size of the Squared Area	SISO	AS	SVD
D=200 m	77.23%	80.71%	74.91%
D=500 m	60.42%	60.32%	49.44%
D=700 m	30.49%	40.07%	23.97%
